# Retinitis Pigmentosa in a Patient With a Homozygous Mutation in the RBP3 Gene: A Case Report

**DOI:** 10.7759/cureus.88992

**Published:** 2025-07-29

**Authors:** Angel Aguayo-Merly, Natalio J Izquierdo

**Affiliations:** 1 Department of Ophthalmology, School of Medicine, Medical Sciences Campus, University of Puerto Rico, San Juan, PRI; 2 Department of Surgery, School of Medicine, Medical Sciences Campus, University of Puerto Rico, San Juan, PRI

**Keywords:** genetic mutation analysis, homozygous pathogenic variant, rbp3 gene, retinal degeneration, retinitis pigmentosa (rp)

## Abstract

Retinitis pigmentosa (RP) is a group of inherited retinal dystrophies characterized by progressive degeneration of the retina, leading to vision impairment. This report presents the case of a 56-year-old female patient with advanced RP caused by a homozygous genetic alteration affecting the RBP3 gene, specifically the c.802 A>T (p.Lys268*) variant. Our patient exhibited classic symptoms of night blindness, bilateral progressive vision loss, and a family history of similar symptoms. Ophthalmic evaluation, including optical coherence tomography (OCT), visual field testing, electroretinography (ERG), fluorescein angiography (FA), and genetic analysis, confirmed the diagnosis of advanced RP. Genetic testing identified the pathogenic homozygous mutation in the RBP3 gene, which, to our knowledge, has not been previously reported in the literature. This case highlights the importance of genetic testing in diagnosing retinal dystrophies, as well as the need for further studies to explore the full spectrum of RBP3-related retinal conditions.

## Introduction

Anneke I. den Hollander first described the association of the retinol-binding protein 3 (RBP3) gene with retinopathies in 2009 [1]. Mutations in RBP3 are linked to a group of inherited retinal dystrophies, including retinitis pigmentosa (RP) and cone-rod dystrophy. Mutations in the RBP3 can be detected in 30% to 80% of patients with RP, depending on the specific type of RP and the technology employed [2,3].

The RBP3 gene is located on chromosome 10 [4]. This gene encodes a protein that plays a crucial role in the transportation of retinoids in the retina [5]. Specifically, RBP3 encodes a protein known as interphotoreceptor retinoid-binding protein (IRBP), which is exclusively expressed by photoreceptors and the pineal gland and is responsible for facilitating retinol transport (movement of vitamin A compounds critical for vision) between the retinal pigment epithelium (RPE) and photoreceptors [6-8]. This binding protein plays a key role in the visual cycle, particularly the regeneration of rhodopsin (renewal of the pigment that enables vision in low light) and photoreceptor function [9,10]. Overexpression of RBP3 has been known to decrease risks for retinal pathologies and even progression to diabetic retinopathy [11].

Mutations in the RBP3 gene disrupt the normal transport of retinol between photoreceptors and the RPE, which is important for photoreceptor function and the visual cycle. These disruptions lead to progressive visual impairment, including symptoms such as nyctalopia, reduced peripheral vision, and a gradual loss of central visual acuity. As the disease progresses, patients may experience reduced color vision, particularly in dim light conditions. Fundus findings, such as retinal pigmentary changes and attenuation of blood vessels, along with electroretinography (ERG) abnormalities, confirm the loss of retinal function. The ERG often shows diminished to absent scotopic response and diminished photopic response (the retina's response to bright light). As the disease advances, macular involvement may also lead to significant central vision loss, further impairing the patient's quality of life.

The age of symptom onset varies depending on the mode of inheritance. Individuals with autosomal recessive RP often become symptomatic in adolescence, whereas those with autosomal dominant forms may not develop symptoms until their 20s to 30s, or even later in some cases, after age 50. Across all forms of RP, the average age at diagnosis has been reported to be approximately 35.1 years [12].

Mutations in RBP3 are typically inherited as an autosomal recessive trait. However, dominant inheritance patterns have been reported [1]. Given the clinical significance of RBP3 mutations, including their potential to cause blindness, progressive vision loss, high myopia, and retinal dystrophy, along with the limited understanding of their diverse presentations, detailed case reports are essential to advance knowledge of this rare retinal disorder. We present the case of a patient with a pathogenic homozygous mutation in the RBP3 gene, offering valuable insights into its clinical features and progression.

## Case presentation

A 56-year-old female patient had a chief complaint of night blindness and bilateral painless progressive vision loss in both eyes. Although she could not recall the exact age of symptom onset, she reported that symptoms began in early adulthood. She had a family history notable for a sister and two brothers with blindness presenting with a similar progressive pattern of vision loss, consistent with hereditary retinal dystrophy. However, genetic testing was not performed on these family members due to a lack of their availability and consent. The patient had no comorbidities reported at the time of evaluation and had no medication regimen. She underwent cataract surgeries in both eyes. 

The patient received a detailed ophthalmologic assessment conducted by a member of the clinical team (author NJI), which also included an optical coherence tomography (OCT); a central threshold 30-2 visual field (VF); a full field electroretinogram (ERG) test; and genetic testing, which included next-generation sequencing (NGS) with a panel for deletions and duplications, performed by Invitae (San Francisco, CA, USA). 

The patient's best corrected visual acuity was 20/100 and 20/200 in the right and left eyes, respectively. Refraction was -5.75 +0.50 x 40 in the right eye and -5.75 +1.50 x 110 in the left eye. Upon slit lamp examination, the patient had pseudophakia in oculus uterque (OU), or both eyes. Intraocular pressure (IOP) was 16 mmHg in the oculus dexter (OD) or right eye, and 17 mmHg in the oculus sinister (OS) or left eye. Fundoscopic evaluation revealed optic disc pallor, arterial attenuation of vessels, and peripheral bony spicules. Her maculae had cystoid macular edema (CME), which was treated with Avastin with no improvement. 

**Figure 1:**
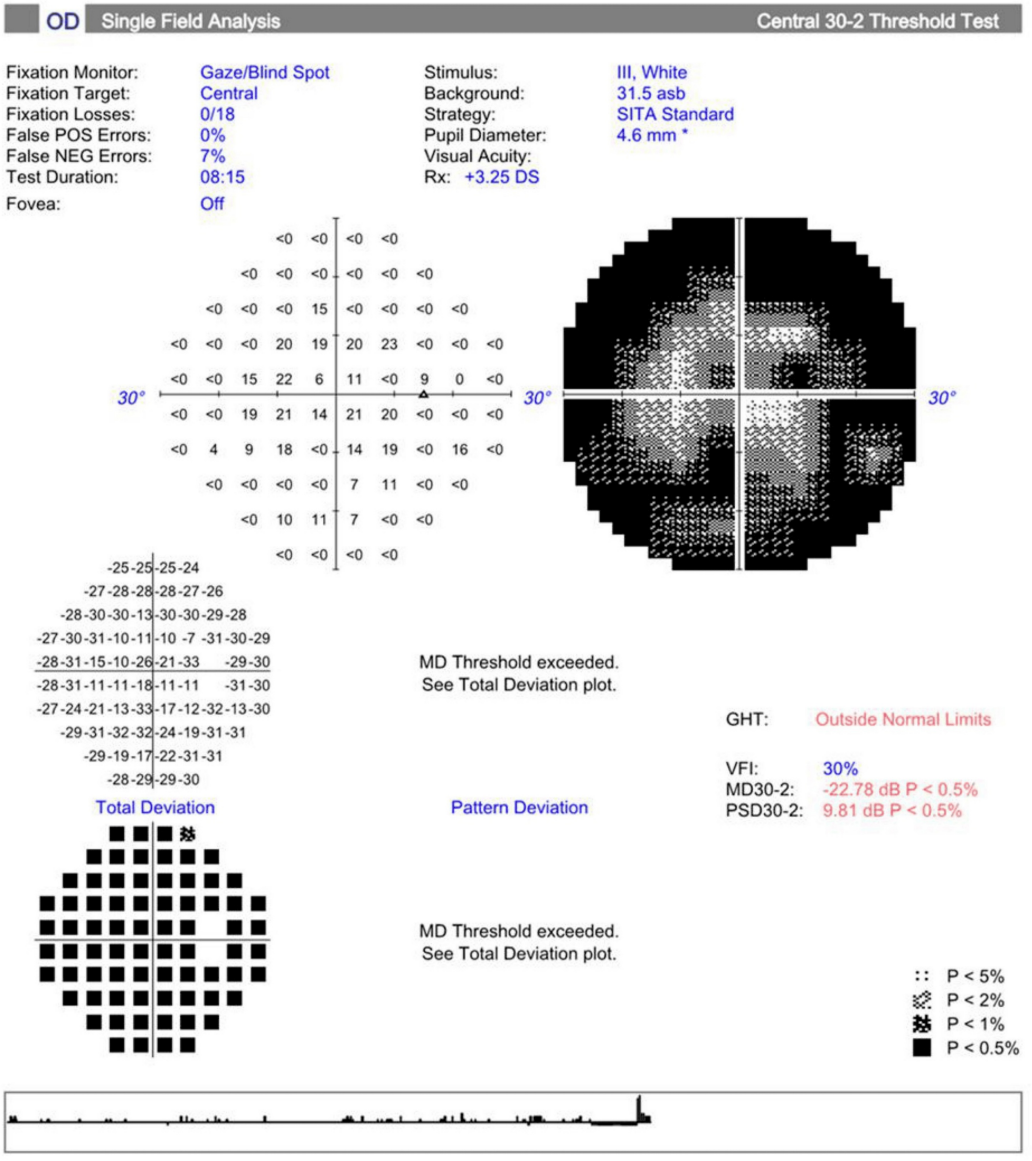
VF testing (30-2, Carl Zeiss) shows decreased mean deviation in the OD OD: *Oculus dexter* or right eye, VF: Visual field, GHT: Gaucoma hemifield test, VFI: Visual field index, MD: Mean deviation, PSD: Pattern standard deviation, DS: Diopter sphere

**Figure 2:**
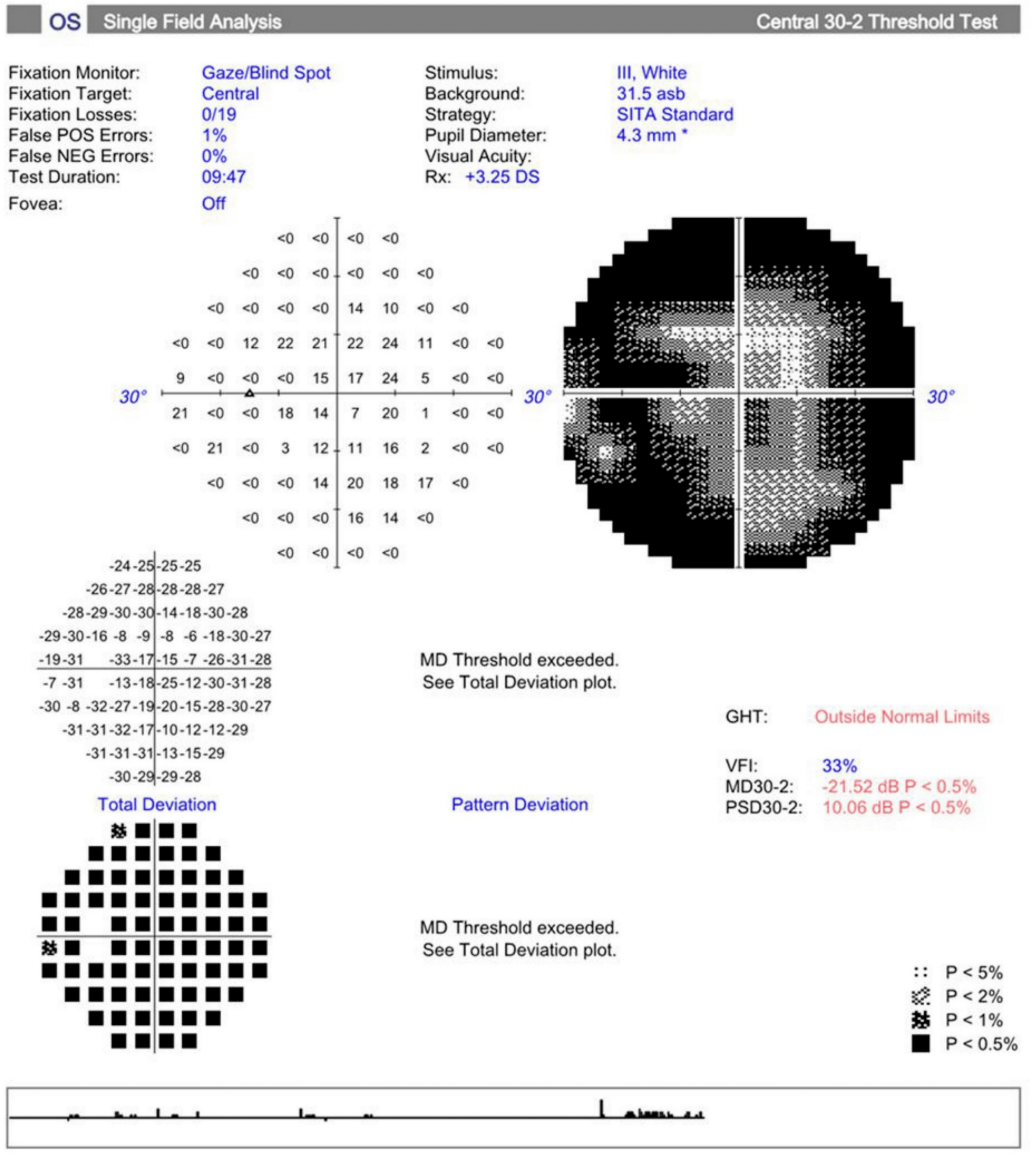
VF testing (30-2, Carl Zeiss) shows decreased mean deviation in the OS OS: *Oculus sinister* or left eye, VF: Visual field, GHT: Gaucoma hemifield test, VFI: Visual field index, MD: Mean deviation, PSD: Pattern standard deviation, DS: Diopter sphere

**Figure 3:**
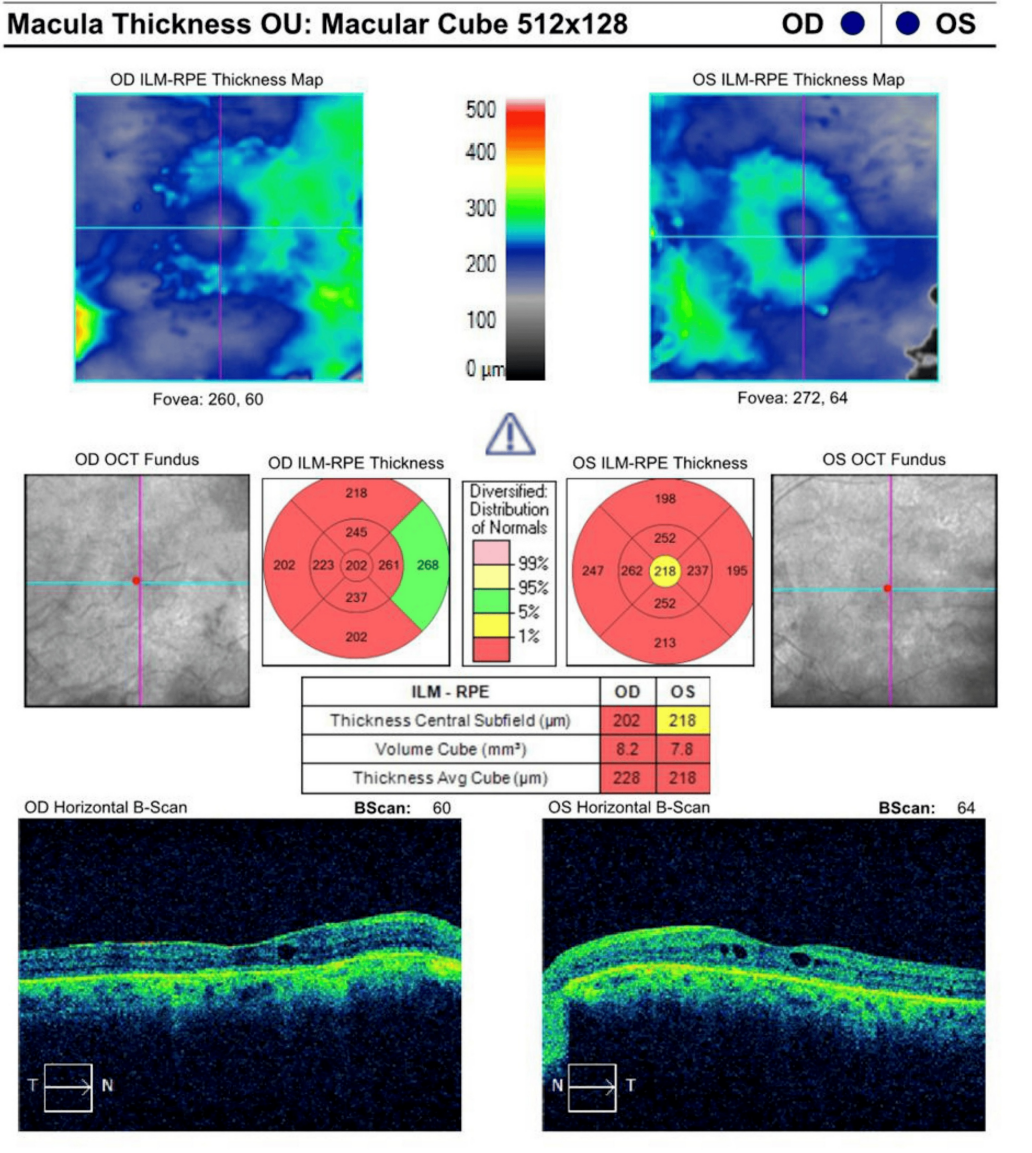
A macular OCT study shows decreased macular thickness and volume bilaterally OCT: Optical coherence tomography, RPE: Retinal pigment epithelium, ILM: Inner limiting membrane, OD: *Oculus dexter* or right eye, OS: Oculus sinister or left eye, OU: *Oculus uterque* or both eyes

As shown in Figure 1 and Figure 2, upon standard automated perimetry using the 30-2 program (Carl Zeiss Meditec Inc., Dublin, CA, USA), patient mean deviation values were -22.78 dB in the OD and -21.52 dB in the OS (both p<0.5). Figure 3 shows the patient's macular OCT with an average thickness of 228 μm OD and 218 μm OS and total macular volumes of 8.2 mm³ and 7.8 mm³, respectively.

**Figure 4:**
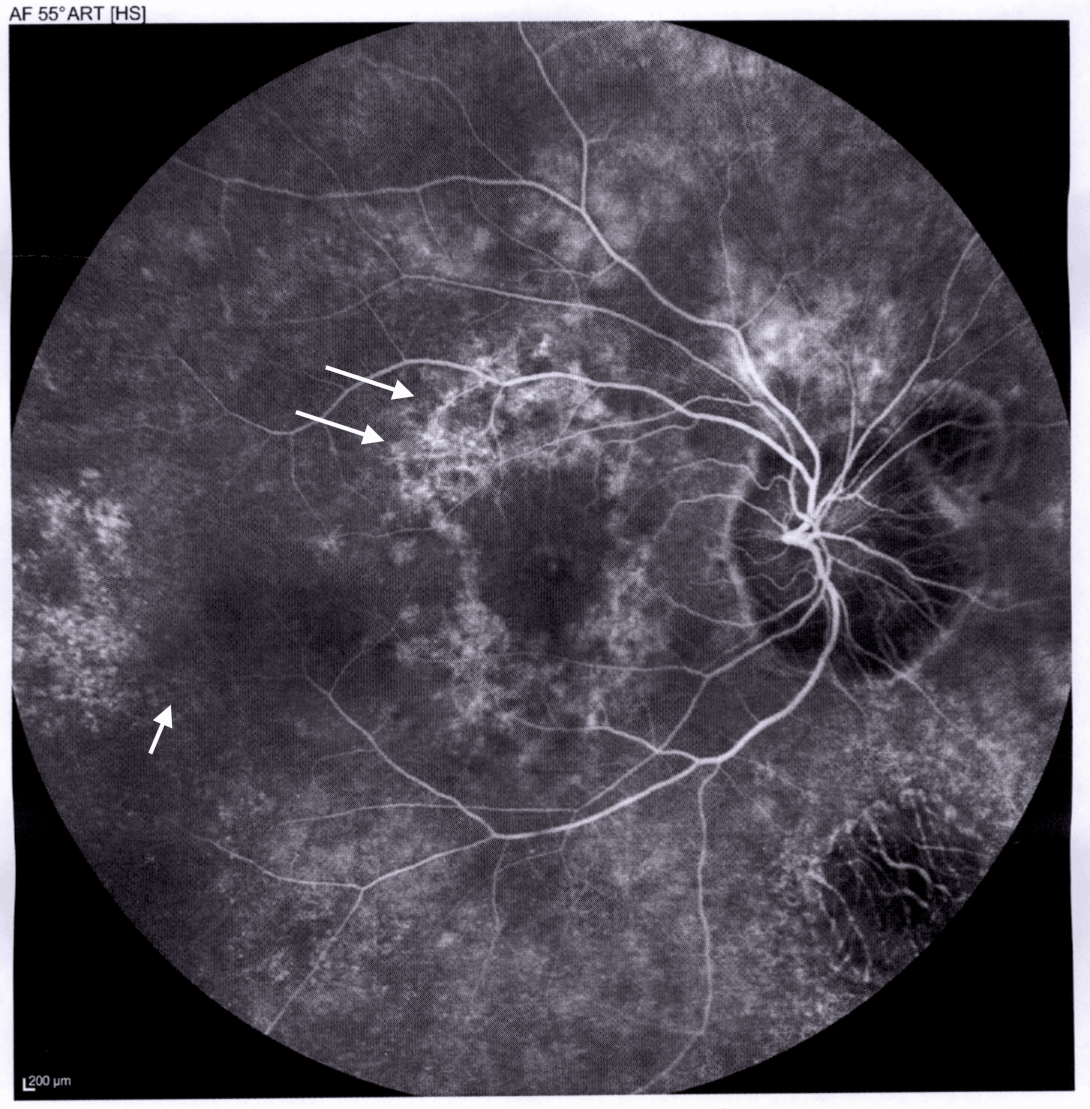
FA of the retina in the OD Late-phase FA shows vascular leakage (single arrow) and capillary non-perfusion (two arrows) in the perifoveal area and temporal retinal periphery. OD: *Oculus dexter* or right eye, FA: Fluorescein angiography

**Figure 5:**
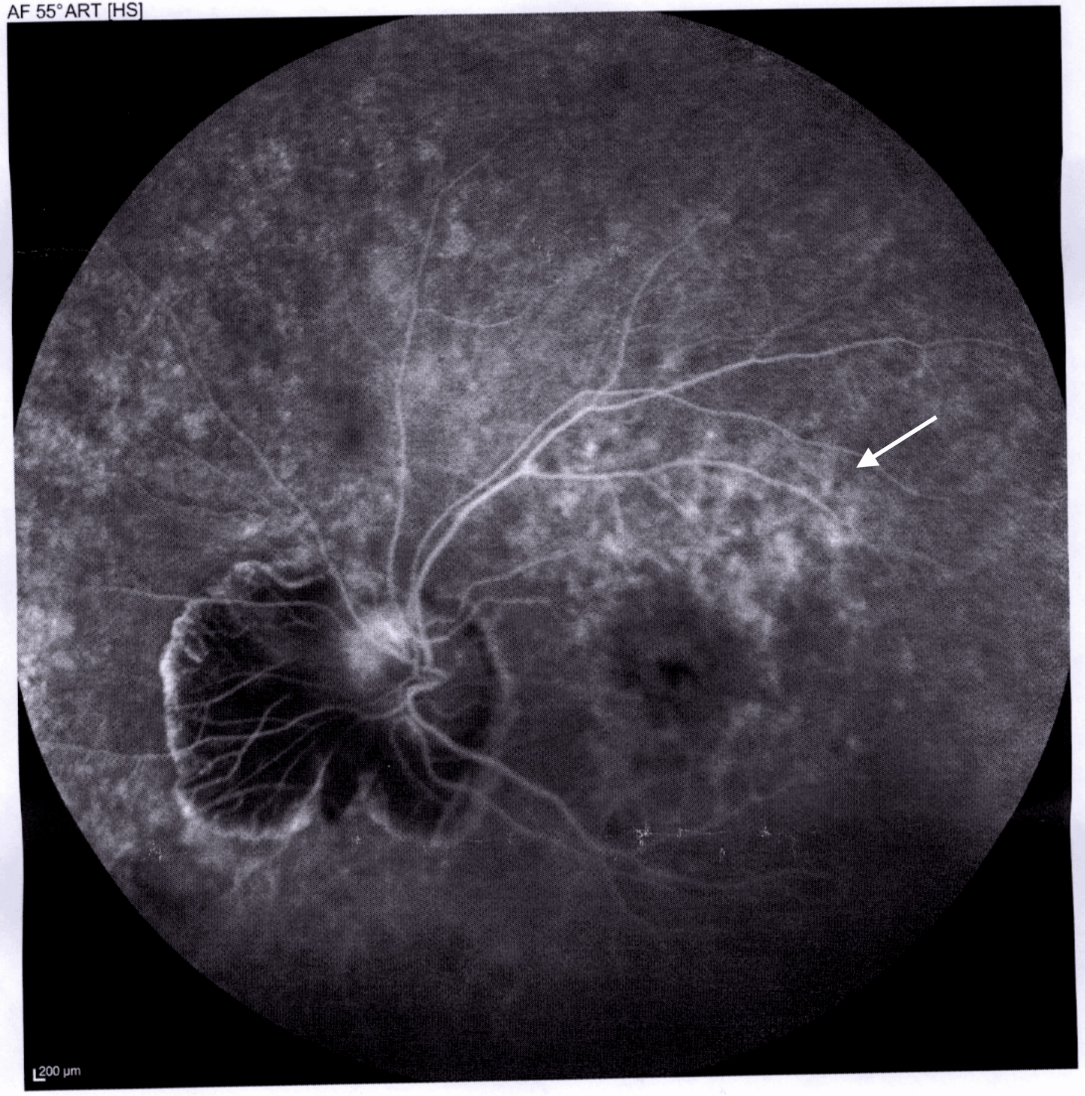
FA of the retina in the OS Late-phase FA shows vascular leakage and capillary non-perfusion in the superior macular area (arrow). OS: *Oculus sinister* or left eye, FA: Fluorescein angiography

Figure 4 and Figure 5 depict fluorescein angiography (FA) images showing vascular leakage and areas of capillary non-perfusion, suggesting retinal ischemia and disruption of the blood-retina barrier. Additionally, the images highlight retinal pigmentary changes, such as vessel attenuation and pigment deposits. 

Upon full-field ERG, scotopic responses were absent, and photopic ERG responses were diminished bilaterally in our patient. A clinical diagnosis of advanced RP was reached. Genetic testing was performed on a saliva sample, and NGS, including analysis for gene deletions and duplications, was conducted using Invitae. Results revealed a homozygous pathogenic variant in the RBP3 gene, specifically c.802 A>T (p. Lys268*).

## Discussion

Retinitis pigmentosa is a group of inherited retinal dystrophies that are characterized by photoreceptor degeneration leading to nyctalopia and gradual loss of peripheral vision, eventually progressing to central vision loss in advanced stages [13,14]. Visual field testing (Figures 1-2) showed significant peripheral vision loss. The patient had a mean deviation of -22.78 dB in the OD and -21.52 dB in the OS. These findings are consistent with rod photoreceptor dysfunction.

The ERG demonstrated absent scotopic responses and diminished photopic responses bilaterally, confirming severe dysfunction in both rod and cone photoreceptors. The OCT (Figure 3) showed thinning of the retinal layers, with an average thickness of 228 μm in the OD and 218 μm in the OS, and macular volumes of 8.2 mm³ and 7.8 mm³, respectively. This thinning of the retina and reduced macular volume suggests progressive retinal atrophy, typical in advanced RP.

Our patient’s FA (Figures 4-5) demonstrated vascular leakage and capillary non-perfusion, indicating retinal ischemia and disruption of the blood-retina barrier, common in advanced RP. Retinal pigmentary changes, such as vessel attenuation and pigment deposits, were also observed, reflecting photoreceptor degeneration.

Mutations in the RBP3 gene lead to isolated retinal dystrophies, that is, without systemic associations. The majority of RP cases are caused by mutations in other genes, with RBP3 accounting for a very small subset [12,15]. Previous studies have reported on patients with homozygous mutations in the RBP3 gene [16]. The individual in this case was found to carry a homozygous disease-causing variant in the RBP3 gene, identified as c.802 A>T (p.Lys268*). Based on our review of the literature, this appears to be the first reported instance of this specific RBP3 mutation in a patient with isolated advanced RP. The RBP3 mutations primarily include missense mutations and nonsense mutations [17]. The most common mutation in RBP3-related RP is unclear due to its rarity. Both missense and nonsense mutations have been reported, such as the homozygous missense p.Asp1080Asn and nonsense mutations p.Tyr510 and p.Glu1152, but no single mutation predominates [1,10]. Nonsense mutations like the one in this case are rare and typically unique to individual families, with no recurrence or overrepresentation compared to missense variants [1,10]. Mutation patterns across ethnicities remain unestablished due to cases coming from varied backgrounds, but there are too few to identify ethnic-specific trends [14]. Our case contributes to the expanding spectrum of RBP3 pathogenic variants and highlights the importance of reporting novel mutations such as p.Lys268*.

The limitation of this case report lies in its focus on a single patient due to the low incidence of mutations in the RBP3 gene, and further studies with larger cohorts are needed to better understand the full spectrum of RBP3-related retinal dystrophies. While genetic testing confirmed a homozygous mutation in the patient, testing was not performed on affected family members (due to their lack of availability and consent), limiting confirmation of how the mutation segregates within the family and the inheritance pattern. Future investigations, including genetic analysis of family members, would enhance understanding of RBP3-related retinal dystrophies.

## Conclusions

Retinitis pigmentosa remains a diagnostic challenge due to its clinical and genetic heterogeneity. Our patient was diagnosed based on OCT showing decreased macular thickness, reduced ERG responses, peripheral field constriction on VF testing, and FA revealing RPE dysfunction. Genetic analysis identified a homozygous c.802 A>T (p.Lys268*) variant in the RBP3 gene, associated with autosomal recessive retinal dystrophies. This case highlights the value of integrating multimodal imaging with genetic testing in RP diagnosis. Further research is needed to clarify genotype-phenotype correlations in RBP3-related retinopathies. Despite the limited data, existing genotype-phenotype evidence indicates that biallelic loss-of-function mutations, including nonsense variants, cause early-onset high myopia and retinal dystrophy with rod and cone dysfunction, often showing minimal fundus changes in childhood. However, the small number of cases restricts definitive correlations.
